# *N^6^*-Methyladenosine and Rheumatoid Arthritis: A Comprehensive Review

**DOI:** 10.3389/fimmu.2021.731842

**Published:** 2021-09-24

**Authors:** Sha Wu, Xiao-Feng Li, Yuan-Yuan Wu, Su-Qin Yin, Cheng Huang, Jun Li

**Affiliations:** ^1^ Anhui Institute of Innovative Drugs, The Key Laboratory of Anti-inflammatory and Immune Medicines, Ministry of Education, Inflammation and Immune Mediated Diseases Laboratory of Anhui Province, School of Pharmacy, Anhui Medical University, Hefei, China; ^2^ Postdoctoral Station of Clinical Medicine of Anhui Medical University, Hefei, China

**Keywords:** *N^6^*-methyladenosine, rheumatoid arthritis, immune cells, autoimmune disease, cancers

## Abstract

Rheumatoid arthritis (RA), one of the most common autoimmune diseases, is characterized by immune cell infiltration, fibroblast-like synovial cell hyperproliferation, and cartilage and bone destruction. To date, numerous studies have demonstrated that immune cells are one of the key targets for the treatment of RA. *N* ^6^-methyladenosine (m^6^A) is the most common internal modification to eukaryotic mRNA, which is involved in the splicing, stability, export, and degradation of RNA metabolism. m^6^A methylated-related genes are divided into writers, erasers, and readers, and they are critical for the regulation of cell life. They play a significant role in various biological processes, such as virus replication and cell differentiation by controlling gene expression. Furthermore, a growing number of studies have indicated that m^6^A is associated with the occurrence of numerous diseases, such as lung cancer, bladder cancer, gastric cancer, acute myeloid leukemia, and hepatocellular carcinoma. In this review, we summarize the history of m6A research and recent progress on RA research concerning m^6^A enzymes. The relationship between m^6^A enzymes, immune cells, and RA suggests that m^6^A modification offers evidence for the pathogenesis of RA, which will help in the development of new therapies for RA.

## Introduction

Rheumatoid arthritis (RA) is a chronic autoimmune disorder characterized by synovial hyperplasia and inflammation, progressive joint destruction, and significant disability (1). Initial lesions in RA include damage to the microvascular system and proliferation of fibroblast-like synovial (FLS) cells that line the synovial membrane of the joint. As the number of synovial cells increases, the cells attach to the articular surface at the edge of the joint, which induces further proliferation and activation of synovial cells. FLS cells are the primary synovial cells, which secrete inflammatory cytokines, chemokines, and metalloproteinases (1). Furthermore, inflammatory cytokines interact with immune cells that are related to RA, such as macrophages, natural killer (NK) cells, dendritic cells (DC), lymphocytes, and mast cells (MCs) (2). Currently, non-steroidal anti-inflammatory drugs and immunosuppressants are commonly used in clinical practice to alleviate the prevent the development of RA. Therefore, further innovations in treatments for RA are needed.

*N* ^6^-methyladenosine of RNA is a methylation modification of adenine (A) at the sixth N, catalyzed by methyltransferase from eukaryotes (3). M^6^A modification has been discovered in various types of RNA, including transfer RNA, ribosome RNA, and even non-coding RNA (4, 5). Through the combination of RNA immunoprecipitation and the m^6^A antibody, RNA-sequencing showed that multiple microRNAs (miRNAs) contained m^6^A modifications (6). MiRNAs target regulated m^6^A-related enzymes and m^6^A-affected miRNA transcriptional regulation; therefore, m^6^A modification is important for the biogenesis and stability of at least some miRNAs. For example, methyltransferase-like 3 (METTL3) affects the binding of DGCR8 to pri-miRNAs, and the absence of METTL3 reduces this binding capacity (4). M^6^A has been applied to immune cells and various immune diseases, with the goal of devising new therapies. So far, researches on the molecular mechanism and function of the m^6^A effectors are abundant (7–10). In this review, we have focused on the potential relationship between m^6^A and RA to explore better methods to treat RA. We highlight several advances in the research of m^6^A in RA, cancers, and immune cells. However, to fully understand the effect of m^6^A on RA *in vivo* and its influence on gene expression, further exploration is needed.

## The Major Enzymes in the m^6^A Pathway

### Writers

**Figure 1:**
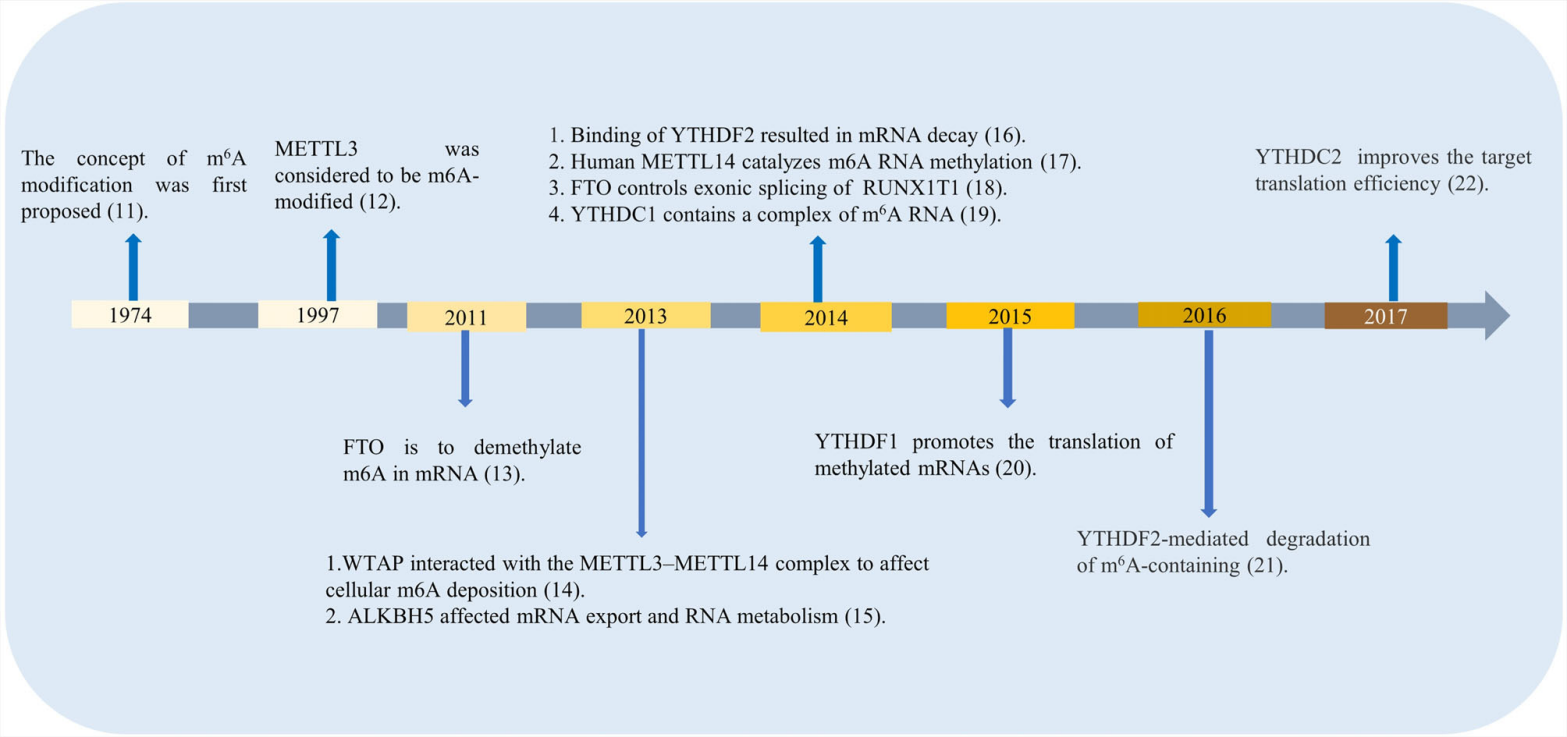
Time for discovery and functions of m6A-related enzymes and proteins.

M^6^A regulators are divided into three types: writers, erasers, and readers. The classifications differ according to the different roles they play in RNA methylation modification, time for discovery, and function of m^6^A-related enzymes and proteins, which are shown in **Figure 1** (11–22). The first type is m^6^A methyltransferase, which promotes m^6^A methylation modification to RNA, and its coding genes are known as writers. The earliest writers discovered are METTL3, METTL14, and wilms’ tumor 1-associating protein (WTAP).

**Figure 2:**
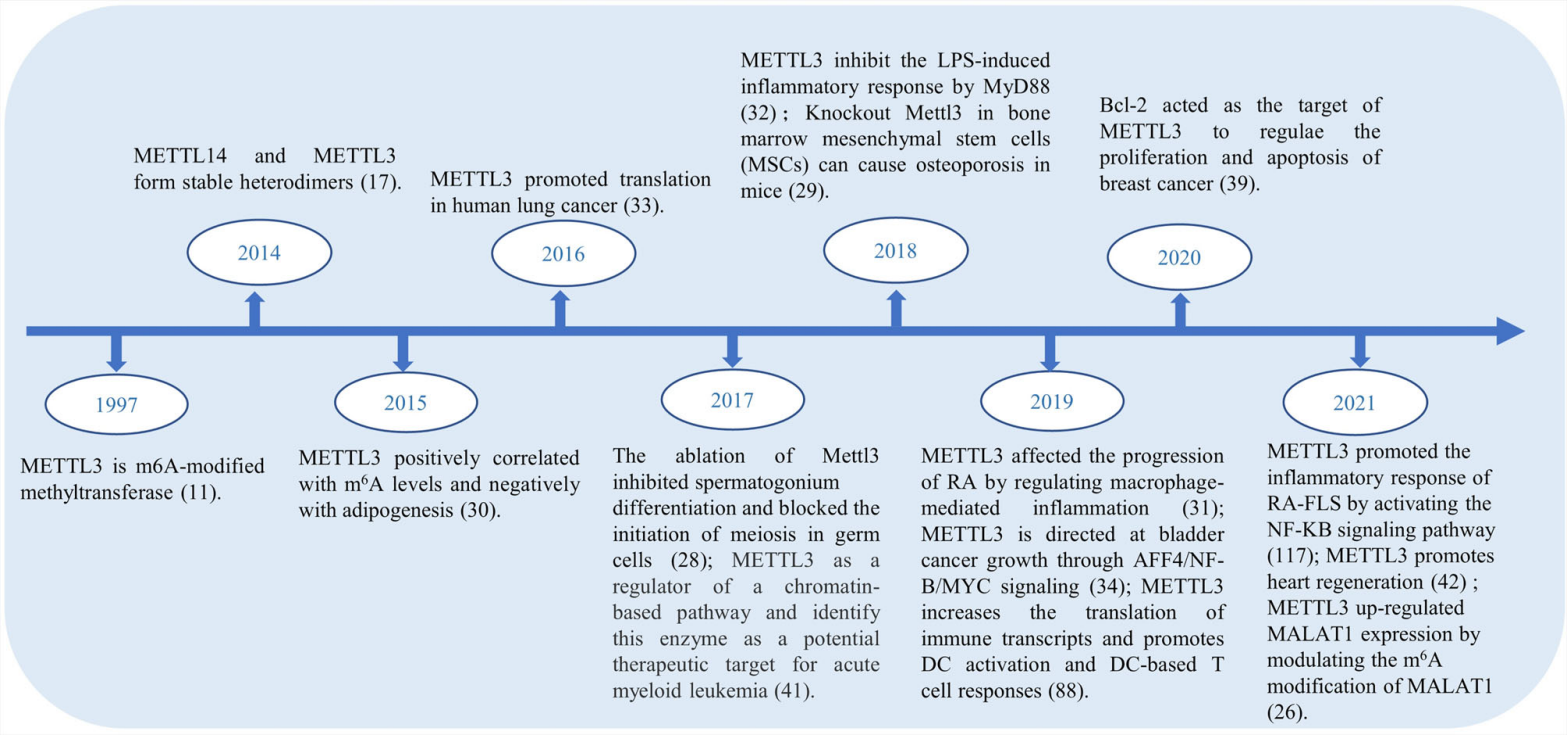
Research progress on METTL3, which is one of the most studied genes in m6A methylation.

In the RNA life cycle of m^6^A, METTL3 has important functions, which include pre-mRNA splicing, nuclear export, translation regulation, mRNA decay, and miRNA processing; moreover, METTL3 also functions to influence epigenetics by regulating and initiating pluripotency (23). Previously, METTL3 was reported as a tumor suppressor because of its upregulating effect on the m^6^A modification (24). METTL3 expression is increased in gastric cancer tissues, hepaticcellcarcinoma (HCC), breast cancer (BC) and mediates the proliferation, metastasis, and colony formation of cancer cells (25–27). In addition, METTL3 plays an unusual role in spermatogonial cells (28), bone marrow mesenchymal stem cells (29), fat mass (30), immune cells and inflammation. **Figure 2** shows the highlights of METTL3 discoveries over time. Recently, studies have indicated that m^6^A methylation modification plays an indispensable role in the innate immune response and antitumor immunity. However, the mechanism of action of METTL3 in RA remains unclear. It has been reported that the expression of METTL3 is significantly elevated in patients with RA, and METTL3 affects the secretion of inflammatory factors in RA through the NF-κB pathway (31). Given the influence on inflammation, cancers, and other diseases, there has been speculation that METTL3 has the potential to treat immune diseases (32). For more information about the expression of METTL3 in diseases and related genes, please refer to **Table 1**.

**Table 1:** m^6^A methylation related to tumorigenesis.

m^6^A methylated related genes	Tissues/cells	Express trend	Regulation of genes	Function	Diseases
Writers:					
METTL3	Human lung cancer cell	up	EGFR, TAZ	Facilitate translation of certain miRNAs (33)	Lung cancer
	Human BCa tissue	up	AFF4, MYC, IKBKB and RELA; pri-miR221/222	Proliferation, invasion, and survival (34, 35)	Bladder cancer
	AGS and MKN45	up	AKT signaling pathway; GFI1	Proliferation and mobility (36, 37)	Gastric cancer
	Human prostate cancer cell	up	GLI1	Apoptosis (38)	Prostate cancer
	Human breast cancer cell	up	HBXIP, miRNA let-7g; Bcl-2	Proliferation and apoptosis (39, 40)	Breast cancer
	AML cell	up	CEBPZ	Enhances its translation (41)	AML
	Bc cell	up	MALAT1, miR-26b	EMT, migration, and invasion (26)	BC
	cardiomyocytes	up	miR-143-3p, Yap and Ctnnd1	Proliferation (42)	myocardial infarction
METTL14	SMMC-7721 and Hep3B cell	down	DGCR8, microRNA 126	Tumor metastasis (43)	HCC
	CRC tissues and Cell	down	miR-375, YAP1	Migration and invasion (44)	Colon cancer
	Mouse hematopoietic stem/progenitor cell	up	SPI1, MYB and MYC	Unknow (45)	AML
	Endometrial tumor	up	AKT signaling; PHLPP2	Proliferation and tumorigenicity (46)	Endometrial
	Nucleus pulposus	up	miR-26a-5p, NLRP3	Viability (47)	Intervertebral disc degeneration
WTAP	Human cholangiocarcinoma cell	up	MMP7, MMP28 and cathepsin H and Muc1	Unknow (48)	Cholangiocarcinoma
	RCC cell lines and tissues	up	CDK2	Proliferation (49)	RCC
	HCC cell	up	ETS1	Proliferation (50)	HCC
	Ovarian tissues, SKOV3	up	MAPK and AKT signaling pathways	Proliferation, migration and Apoptosis (51)	HGSOC
	Human bladder transitional cell cancer tissues	up	Unknow	Unknow (52)	Bladder cancer
	Human Pancreatic ductal adenocarcinoma (PDAC)	up	CCHC-type zinc finger nucleic acid binding protein	Promote the translation (53)	(PDAC)
RBM15	32DWT18 myeloid precursor cell line	up	HES1	Hematopoiesis (54)	
Erasers:					
FTO	MLL-rearranged AML cell lines	up	ASB2 and RARA	cell differentiation (55)	AML
	HCC tissues and HCC cell lines	up	PKM2	Accelerate the translated production (56)	HCC
	NSCLC cell	up	USP7	Colony formation (57)	Lung cancer
	MDA-MB-231 cell and MCF-7 cell	up	BNIP3	Growth retardation、metastasis (58)	Breast cancer
	Human umbilical cord-derived mesenchymal stem cells	up	HUC-MSCs-derived exosomal miR-627-5p	Glucose and lipid metabolism (59)	Non-alcoholic fatty liver disease
	Melanoma cell	up	PD-1,CXCR4 and SOX10	Increased RNA decay (60)	Melanoma
ALKBH5	Human breast cancer cell lines	up	NANOG	Tumor initiation (61)	Breast cancer
	Human GSC cell lines	up	FOXM1	Nascent transcripts (62)	Glioblastoa
	SGC-7901, BGC-823, and GES-1 cells	up	NEAT1	Invasion and metastasis (63)	Gastric cancer
Readers					
YTHDC1	Oocyte	-	Unknow	Increases translation efficiency (64)	
YTHDC2	Human colon adenocarcinoma cell lines	up	HIF-1α	Metastasis (65)	Colon cancer
YTHDF1	HeLa cell	up	Unknow	mRNA translation (20)	
	hepatocellular carcinoma cell	up	Unknow	Cell cycle progression metabolism (66)	HCC
	Colorectal tissues	up	Wnt/β-catenin pathway	Unknow (67)	CRC
YTHDF2	Hematopoietic stem cell		Tal1 mRNA	Decay (68)	
YTHDF3	Human colorectal cell line	up	lncRNA GAS5, YAP	Promote Inc RNA degradation (69)	Colorectal cancer

METTL14 is a pseudo methyltransferase that stabilizes METTL3 and recognizes target RNA (45). The combination of METTL14 and METTL3 promotes the identification of m^6^A by METTL3 to some degree. The closed connection between full-length polypeptide methylation with point mutations in the catalytic base sites of METTL3 and METTL14 indicates that METTL3 is the only subunit with catalytic activity *in vitro* (70). There is a wide range of beneficial interactions between METTL3 and METTL14, which are important for stabilizing the structure of both domains as well as for interdomain coordination. In contrast to METTL3 studies, few studies have been published on METTL14 alone. As an essential component of the m^6^A methyltransferase complex, METTL14 is highly expressed in hematopoietic stem/progenitor cells (HSPCs), acute myelogenous leukemia (AML) cells, and pancreatic cancer (45, 71). Furthermore, studies have revealed that METTL14 depletion is dependent on mTOR signaling-induced autophagy (30).

WTAP is an m^6^A methyltransferase complex that is found in mammals. In the absence of WTAP, the RNA binding capacity of METTL3 is significantly weakened (72). This suggests that WTAP regulates the recruitment of the m^6^A methyltransferase complex to the mRNA target and affects its binding capacity. WTAP functions without methylation activity, but it interacts with the METTL3 and METTL14 complexes to significantly affect cellular m^6^A deposition. Furthermore, WTAP participates in crucial cellular processes, such as regulation of the cell cycle (14, 73), cell proliferation, and cell apoptosis. Growing evidence has shown that WTAP is related to the malignant potential of tumor cells, is clearly upregulated in HCC tissues (50), and is responsible for the migration ability of the SKOV3 cell line (51). These results demonstrate that WTAP plays a vital role in proliferation and invasiveness abilities.

Similar to other RNA-binding motifs (RBM) proteins, RBM15 combines with RNA through spliceosomes to regulate splicing, translation, and stability. At present, RBM15 has been studied widely in various blood diseases, such as chronic myelogenous leukemia (CML), acute megakaryocytic leukemia, and T−cell acute lymphoblastic leukemia. Yang et al. found that patients with chronic and accelerated-phase CML had a significantly lower mRNA expression level of RBM15 than that of patients with blast-crisis CML. Moreover, the RBM15 protein may affect the growth of RBPJ-mediated CML cells through Notch signaling (25, 74). Rbp-Jk is one of the major canonical transcriptional effectors in the Notch signal pathway.

### Erasers

The second type of protein is an m^6^A demethylase called erasers, which remove the m^6^A methylated group from RNA. The most common erasers are the fat mass and obesity-associated protein (FTO) and alkylation repair homolog protein 5 (ALKBH5).

Originally, FTO was thought to be a protein that regulates body weight and obesity. Overactivation of FTO can increase food intake, which leads to obesity. The FTO controls mRNA splicing by inhibiting SRSF2 (a RNA splicing factors) binding at splice sites (18). However, when FTO was recognized as an RNA demethylase, it was found to have additional functions for controlling various aspects of biological processes. FTO removes the m^6^A modification and modulates the stability of mRNA, which ultimately leads to the alteration of pathogenesis in various types of cancer (55, 75). In some tumor cells, FTO is critical for their immune escape. FTO-deleted made melanoma cells sensitive to interferon therapy and anti-PD-1 therapy (60). FTO and programmed death-ligand 1 (PD-L1) were both high expressing in colon cancer cells, and FTO regulate the expression of PD-L1 (76). In arsenic-associated diseases, FTO is upregulated. While FTO-deleted promoted autophagy and inhibited arsenic- associated tumorigenesis (77). Some observations of FTO suggest that it plays a crucial role in the proliferation and apoptosis of some cancer cells, also glucose and lipid metabolism (59, 78, 79). Applying this biological function of FTO to inflammatory immune cells could inhibit the release of inflammatory cytokines and alleviate inflammatory disease.

ALKBH5 is known for regulating transcriptional modification in numerous human malignancies and maintaining mRNA stability (62). ALKBH5 is primarily involved in the demethylation of m^6^A modification and exerts its functions during the regulation of mRNA nucleation and other metabolic processes in the development of sperm. The functions of m^6^A modification are involved in the regulation of IncRNA to affect the development of cancers (80). In addition, ALKBH5 regulated PD-L1 mRNA in intrahepatic cholangiocarcinoma (ICC). The lack of ALKBH5 decreased the expression of PD-L1 on monocytes-macrophages by a YTHDF2-dependent manner (81). Also, the deletion of the m6A demethylase Alkbh5 sensitized tumors to cancer immunotherapy (82). It extended the cognition of ALKBH5 in tumor immune microenvironment and immunotherapy.

FTO and ALKBH5 inhibit proliferation by regulating cell migration, invasion, and metastasis in some cancer cells. However, few studies on these proteins have investigated immune diseases. If proliferation and migration of synovial cells are reduced by FTO and ALKBH5, the onset of the inflammatory response in RA could be delayed. Although the role of m^6^A erasers has not been extensively explored in immune diseases, it has promise as a valuable research direction for FTO and ALKBN5.

### Readers

The final group of proteins plays a specific role by binding to the m^6^A methylation site in RNA, and their coding genes are called readers. There are five YTH domain-containing proteins in the human genome: YTHDC1, YTHDC2, YTHDF1, YTHDF2, and YTHDF3. YTHDF1 enhances mRNA translation and protein synthesis by interacting with initiation factors (20). YTHDF2 induces degradation of transcripts by selectively binding to and recruiting m^6^A-modified mRNA to mRNA decay sites (16). YTHDF3 enhances RNA translation by interacting with YTHDF1 and promotes RNA degradation by associating with YTHDF2 (83).

YTHDF1 not only participates in mRNA translation, which directly targets YTHDF1 or binds to translational initiation factors in some cancer cells but is also involved in neoantigen-specific immunity. In classical DCs, the absence of YTHDF1 enhances the cross-presentation of tumor antigens and the cross-priming of CD8+ T cells *in vivo* (76). Firstly, YTHDF1 distinguishes between m^6^A-related mRNAs in DCs that encode lysosomal proteases. The binding between m^6^A-marked mRNAs and YTHDF1 then boosts lysosomal protease translation, which suppresses the cross-presentation of engulfed tumor neoantigens. Furthermore, PD-L1 expression was increased in tumor cells from Ythdf1^−/−^ tumor-bearing mice, anti-PDL1-treated on Ythdf1^−/−^ tumor-bearing mice made tumor disappeared, the simultaneous depletion of PD-L1 and YTHDF1 was beneficial to the improvement of tumor immune microenvironment (76). This process is a novel mechanism of immune evasion to elude immunosurveillance.

YTHDF2 destabilizes key gene transcripts in certain biological processes. Furthermore, the knockdown of YTHDF2 leads to reductive proliferation of lung cancer cells (84). However, YTHDF2 is specifically downregulated in HCC cell lines under hypoxia culture conditions, and the overexpression of YTHDF2 unexpectedly inhibits the growth and proliferation of HCC cells (85). There are two clearly opposing effects on the regulating process of YTHDF2, and further research into its mechanisms is required. In addition, Yu et al. (78) discovered that the YTHDF2 expression level is upregulated in macrophage RAW264.7 cells after stimulating with LPS. Moreover, the knockout of YTHDF2 expression contributed to the downregulation of mRNA proinflammatory factor levels. The function of YTHDF2 in preventing an excessive inflammatory response offers a potential target and a new perspective for inflammation in the treatment of immune diseases (86).

YTHDF3 has been proposed to be the first reader protein to interact with m^6^A-modified transcripts in the cytoplasm (83). Denise and colleagues (59) found that knocking out YTHDF3 in human CD4+ T cells increases the risk of infection, and YTHDF3 acts as a limiting factor for human immunodeficiency virus (HIV). In this study, YTHDF3 proteins were mixed with HIV particles in a nucleocapsid-dependent manner, allowing the m^6^A reader protein to limit infection in the new target cell at the reverse transcription step, which reduced viral infectivity in the next cycle of infection (87). In studies related to YAP signaling pathways, YTHDF3 induces GAS5 decay by recognizing m^6^A modified GAS5 through a negative feedback loop between lncRNA, GAS5, and YTHDF3. The functional link between the YAP signaling pathway and the m^6^A modification offer a promising approach for other research (69).

YTHDC1 is located in the nucleus, whereas YTHDF2 and YTHDC2 are found in the cytoplasm. YTHDC1 affects the processing of pre-mRNA transcripts in germ cells and directly affects the maturation of oocytes (64). YTHDC2 is of great importance in spermatogenesis, where it selectively binds to m^6^A at its consensus motif. Genital morphology mice with a knockdown of the YTHDF3 gene had smaller ovaries and testes in females and males, respectively. It is possible that high expression of YTHDC2 heightens the translation efficiency of its targets during spermatogenesis.

### State of the Art Knowledge: How m^6^A Affects Immune Cells

Wang et al. demonstrated that DC activation and function for promoting CD4+ T cell proliferation decline following inhibition of the m^6^A modification. During DC maturation, METTL3 catalyzes m^6^A in CD40, CD80, and Tirap, which facilitates DC activation by increasing translation efficiency and function for promoting T cell activation (88). DCs are specialized antigen-presenting cells (APCs) that are linked to innate and adaptive immune responses (89). These studies indicate that m^6^A plays a vital and flexible role in the innate immune response and antitumor immunity.

M^6^A has a significant influence on the homeostasis and differentiation stability of T cells. Li et al. demonstrated in mice that if the m^6^A protein, METTL3, is absent from T cells, T cell homeostasis and differentiation are easily disrupted. The number and characteristics of immune cells in mice with a conditional knockout of METTL3 were detected under a stable state: abnormal T cells were found in the spleen and lymph nodes (LNs), and there was an increase in the number of naive T cells in the LNs. In non-small-cell lung cancer, circIGF2BP3 inactivated cocultured CD8^+^T cells and METTL3 depended on YTHDC1 to participate in the methylation modification of circIGF2BP3 (90). Subsequently, Li et al. found that, through the TCR-dependent T cell differentiation system, TH1 and TH17 cells in METTL3-deficient naive T cells were decreased, TH2 cells were increased, and Treg cells remained unchanged (91). M^6^A modification plays an important role in the differentiation of CD4^+^ T cells. Previous studies have shown that m^6^A induces the degradation of soc mRNA in response to IL-7 signals to reprogram the proliferation and differentiation of naive T cells, which indicates a novel T cell homeostasis mechanism and signal-related induction of mRNA degradation (92).

As an important part of innate immunity, polarization changes between M1 and M2 phenotypes of macrophages regulate various physiological and pathological states. Recent findings have suggested that the m^6^A catalytic enzyme, METTL3, promotes M1 macrophage polarization to play a pro-inflammatory role. Mechanistically, METTL3 directly methylates the mRNA of STAT1, which is a critical transcription factor for priming proinflammatory macrophages, and enhances its mRNA stability; thus, upregulating STAT1 expression and facilitating M1 macrophage polarization (93). Therefore, METTL3-mediated m^6^A methylation in macrophages may serve as a potential anti-inflammatory target in the treatment of inflammatory diseases. In a fascinating new study, Zhang and colleagues identified a novel mechanism by which METTL3 acts during oxidized low-density lipoprotein (oxLDL)-induced monocyte inflammation. In human monocyte PHT-1 cells, METTL3 and YTHDF2 jointly affect the state of mitochondria and energy metabolism, thereby enhancing the inflammatory response of monocyte-macrophage (94). Basing on this research, we can open up various ideas about the pathogenesis of inflammatory and immune diseases.

Type I interferon produces an immune response to viral infection. Winkler and colleagues proposed a new approach for m^6^A targeting of IFNB mRNA to regulate the type I interferon response, which limits the duration of the antiviral response (95). After viral infection, depletion of METTL3 leads to elevated levels of type I interferons because m^6^A modification of interferon transcripts regulates their decay rates. Further studies on the role of m^6^A modification in the immune system may provide new therapeutic options for inflammatory and infectious diseases.

## Translational Potential

### The Potential Link Between RA and Immune Cells

In autoimmune diseases, the immune response is initiated by local innate immune cells that are exposed to external antigens or autoantigens. With the advances in surgical techniques, such as synovial excision and surgical correction, Janossy et al. found that the number of HLA-DR+ macrophages is greater in RA synovial tissue (96). In RA, monocytes and macrophages, as specialized APCs, stimulate the response and infiltration of inflammatory cells and play a considerable role in joint destruction (97). B cells produce immunoglobulins, inflammatory cytokines, rheumatoid cytokines, and other factors that promote the positive feedback regulation of macrophages.

In the inflamed RA joint, tumor necrosis factor (TNF) is produced primarily by macrophages, and TNF inhibitors are effective for the treatment of RA (98). Secretion of cytokines and chemokines perpetuates the inflammatory response by recruiting additional innate immune cells, such as monocytes and neutrophils, and also by inducing T cell differentiation (99). Moreover, abnormally activated macrophages and T cells migrate to the joint cavity and are stimulated by the activated synovial cells to aggravate the inflammatory response in the synovial microenvironment, which leads to synovial inflammation, synovial hyperplasia, and cartilage in the joint tissues.

In the early 1990s, macrophage gene expression was found to vary under different media stimulation (IL-4/IFN-γ/LPS). In 2000, Mills et al. (65) discovered M1-M2 polarization because of arginine metabolism in macrophages. They further proposed that the M1-M2 dichotomy is an intrinsic property of the transition of macrophages from inflammation to healing. However, findings of subsequent studies have deviated from the initial concept of Mill et al., although the definition and distinction of M1-M2 remain controversial. The inconsistencies in macrophage activation and biomarkers introduce challenges in the study of M1-M2 polarization of macrophages. Kung et al. proposed proinflammatory cytokines and anti-inflammatory cytokines as features that distinguish M1 macrophages (CD80+) from M2 macrophages (CD163+) (100). Although there is little evidence for M1-M2 polarization, we recognize that is involved in RA. Indeed, the mass proliferation of synovial cells is typical of RA and is closely related to macrophages. Therefore, a treatment targeting macrophages in the articular cavity may have a positive effect on the disease by reducing inflammation and joint damage.

NK cells are natural immune effector cells with a direct killing function and are particularly important in immune cells because they eliminate viral infections and secrete proinflammatory cytokines. Li et al. conducted an integrative pathway association analysis of RA using genome-wide association studies (GWAS) summary data of 25 RA-associated pathways for NK cells (2). A large number of NK cells were found in the synovial fluid of RA patients, which is considered an important factor for bone destruction (101). In a mouse model of arthritis, exhaustion of NK cells before constructing the mouse model provided almost complete protection of bones and joints from erosion (102). In the synovial fluid of 15 patients with inflammatory arthritis, NK cells expressed high levels of CD56 and low levels of CD16 was significant increase. They further showed that proinflammatory factor IL-12, in combination with IL-15, is effective in stimulating NK cells to express IFN (103). However, IFN itself can activate macrophages and regulate the transcription of genes within macrophages, which suggests that NK cells could interact with articular macrophages/monocytes to produce more proinflammatory cytokines (103). In addition, RBM15 regulates the Notch signaling pathway (71). It is known that the Notch signaling pathway is related to the number of NK cells to some degree, and a variety of excitatory and inhibitory receptors are inherently expressed on the cell surface, which monitor malignant cells (104). After RBM15 regulates the Notch signaling pathway, the Notch signaling or canonical transcriptional effectors affect immune cells, such as NK cells. Eventually, the immune cells operate throughout the entire immune system by linking the immune system. This generates natural immunity and activates acquired immunity to maintain the body’s normal immunity status. Taken together, interfering with the activation of NK cells may provide a basis for therapeutic strategies.

Autoreactive T cells play an important role in many autoimmune diseases. There are various types of T cells, such as the T helper cell and nonclassical T helper cell subsets, such as Th0, Th1, Th2, Th17, Th1/17, CCR4, CCR6, and CXCR3 (105). Yasuo et al. found that the frequency of memory CXCR4(+) CD4(+) T cells was significantly associated with RA severity (91). Furthermore, autoreactive T cells have been shown to be critical in many models of autoimmune diseases, such as collagen-induced arthritis, non-obese diabetic mice, and experimental autoimmune encephalitis (106). The PD-1/PD-L1 is one of the pathways for tumors to evade the immune system by challenging T cell tolerance, T cell exhaustion, enhancing immunosuppressive Treg cell function and inducibling co-stimulatory molecule (107, 108). Thus, T cell immunity as a target for more precise immunotherapy interventions for human autoimmune diseases is likely to be a more selective way to treat RA.

In addition to autoantibody production, B cells are efficient APCs, which allow T cell activation. In some RA patients, B cells have been found to differentiate into plasma cells that produce rheumatoid factor and anticyclic citrullinated peptide antibodies (109). Rituximab is primarily used to deplete the ability of B cells and alleviates RA in patients who have TNF inhibitor failure. Inspired by clinical experience with rituximab, various approaches for investigating the pathogenic function of B cells for RA are popular. Currently, direct targeting of B cells in rheumatic diseases is divided into four broad categories: CD19, CD20, CD22, and CD52. Target-specific surface molecules effectively deplete B cell populations, which control immunity and B cell activation (110).

DCs are thought to play a crucial role in immunopathogenic responses, which result in the establishment of chronic proliferative synovitis and joint destruction in RA (111). Frances demonstrated that RA synovial fluid contains specific subsets of myeloid progenitor cells, which could differentiate into mature DCs in the correct microenvironment (112). It may ultimately be possible to provide some basis for the heterogeneity of the immune response in RA. Huang et al. demonstrated that etanercept (ETN), which is a TNF antagonist, prevented arthritis development through the regulation of DC actions. ETN reduces the ability of DC to migrate to local LNs and regulates the quantity of T and B cells to change the composition of LN cells. Moreover, it has been shown that TNF-α blockade has a significant impact on DC maturation and migration, which contributes to the immune regulatory effects in RA patients (113). There is evidence that mature DCs present in the RA joint mediate immunopathology. These studies suggest that RNA modification of m^6^A has a potential function between DCs and RA.

MCs are important immune cells in the mammalian body and affect the pathogenesis of. MCs recognize and respond to various antigens, secrete various chemokines, recruit other immune cells, and cause local tissue inflammation, neovascularization, and tissue remodeling in the body (114). They exist in normal and RA synovium. However, the number of MCs in the synovium in RA patients is higher, and active RA patients show higher MC infiltration than those at the end stage of the disease (115).

Numerous studies have shown that immune cells play a role in the pathogenesis of RA. First, some immune cells secrete chemokines and growth factors and subsequently, infiltration of other immune cells into synovial cells occurs. Second, immune cells can act as APCs to stimulate the production of various other immune cells. Third, in the early stage of RA, synovial cells have a small expansion area. Through the suppression of specific immune cells, combined with the patients’ age, sex, diet, constitution, and other factors to give appropriate treatment, it is a great approach to fully improve the treatment effect. Finally, whether immune cells promote angiogenesis in the synovium remains to be elucidated.

## The Gap in Knowledge for Future RA Research

So far, the presence of m^6^A methylation in RA has been poorly studied. Wang and colleagues showed that in RA patients, the expression of METTL3 is markedly increased in peripheral blood mononuclear cells (PBMCs); however, other RNA m^6^A methyltransferases have no obvious (31). In vitro experiments have shown that by stimulating LPS, total m^6^A content can be enhanced by upregulating METTL3 in pTHP-1 macrophages and that METTL3-related m^6^A modification is associated with cytokines IL-6 and TNF-a (31). In other words, METTL3 attenuates inflammation through the NF-κB pathway in RA, which could be one approach to treat RA. In addition to the mechanism of action of METTL3 in inflammatory immune macrophages. METTL3 had also been reported in synovial cells recently. METTL3 promoted the inflammatory response of RA-FLS by activating the NF-KB signaling pathway (116). The mRNA expression of ALKBH5, FTO and YTHDF2 in RA patients’ PBMCs was significantly decreased (117), it also provided novel insights into recognizing the pathogenesis of RA.

**Figure 3:**
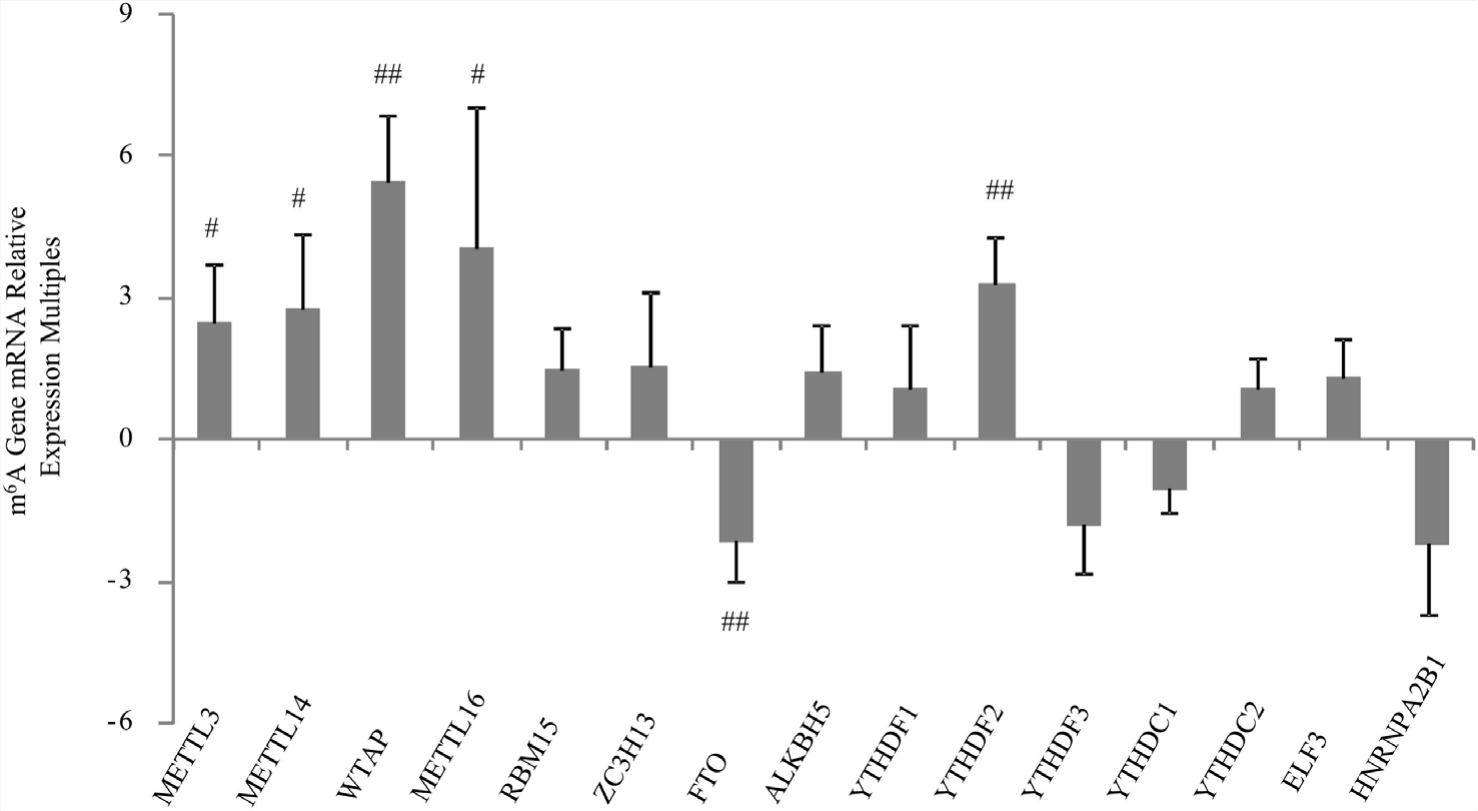
mRNA expression of m^6^A-related enzymes was quantified using qRT-PCR in the synovial tissues of RA and osteoarthritis patients. The histogram represents multiple expressions of m6A-related enzymes in RA. The expression in osteoarthritis is 1. Values represent means ± standard deviations. ^##^ *p* < 0.01 *vs.* OA; ^#^ *p* < 0.05 *vs.* OA.

The main symptom of severe RA is synovial hyperplasia. Inhibiting the infinite proliferation of synovial cells is an important treatment method to curb the development of RA. There are few studies on the treatment of RA from the perspective of m^6^A methylation. However, our previous study showed that m^6^A-regulated methyltransferase, demethylase, and binding protein genes are altered in the synovial tissues of patients with RA. Control tissues were synovial tissues of patients with osteoarthritis. Using qRT-PCR, we found that METTL3, METTL14, WTAP, METTL16, and YTHDF2 were significantly upregulated, and FTO was significantly downregulated, whereas the others did not change significantly. Moreover, the expression of WTAP was significantly upregulated in the RA synovial membrane (**Figure 3**). The histogram in **Figure 3** represents multiple compared with osteoarthritis, and osteoarthritis is 1.

## Discussion

**Figure 4:**
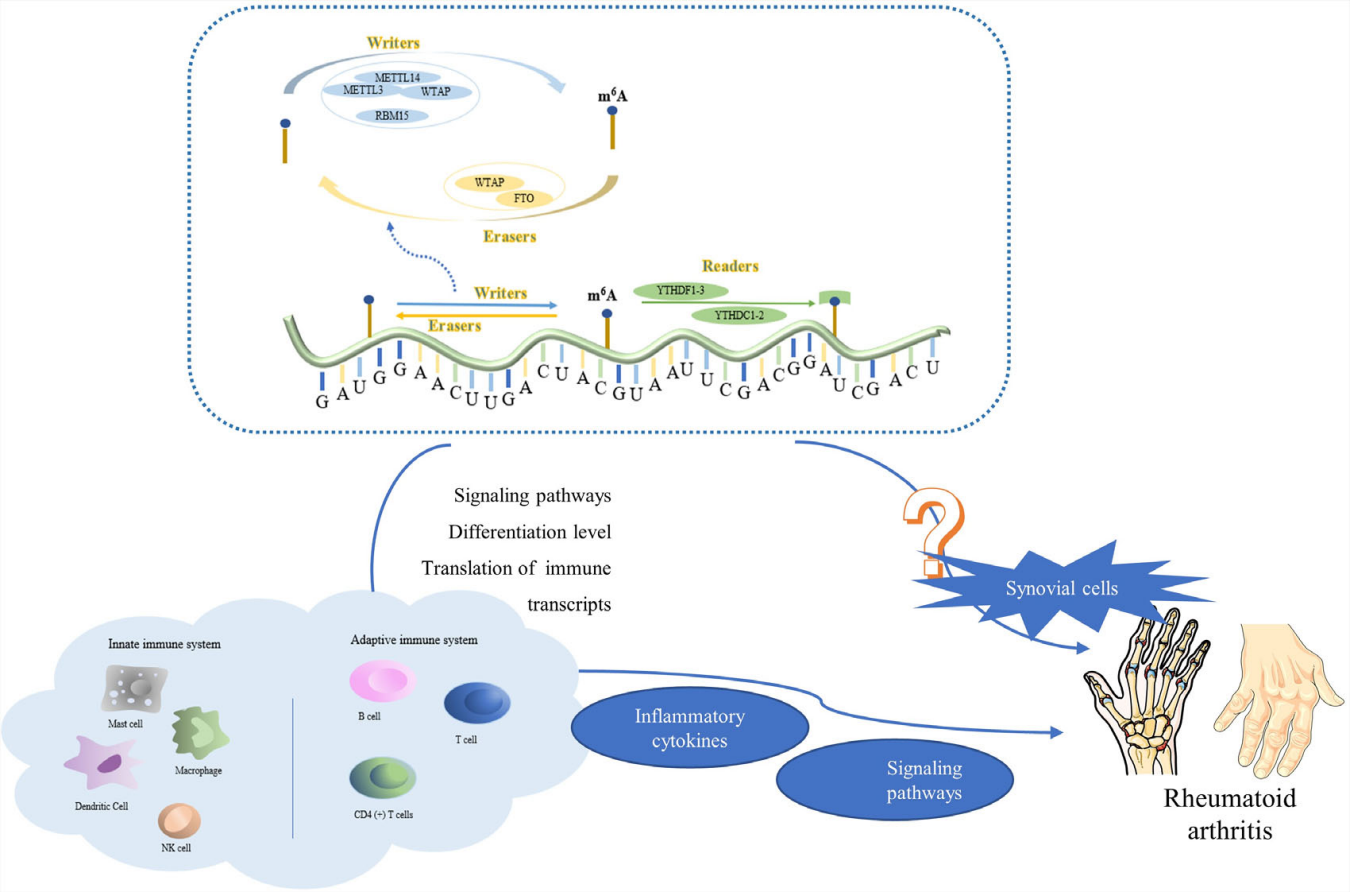
The complexes formed by METTL3, METTL14, WTAP, and RBM15 are common writers; those formed by ALKBH5 and FTO are common erasers; and those formed by YTHDC1, YTHDF1, and YTHDF3 are common readers. M^6^A methylation is involved in a wide range of biological regulatory processes in the innate and adaptive immune systems, such as adjustment of signaling pathways, differentiation, translation of immune transcripts. It controls the production of inflammatory factors, inflammation-related signaling pathways, and other genes that have a direct impact on RA. It is worth noting that, m^6^A methylation may also directly affect synovial cell proliferation and tumor tissue, which may be a potential discovery in future RA research.

M^6^A methylation belongs to one of the many families of RNA epigenetic modification. Based on the current understanding of its relationship with tumors, m^6^A methylation does not have a “good or bad” effect on tumor cells and promotes or inhibits tumor cells by regulating the mRNA expression level of related oncogenes or tumor suppressor genes. Extensive research on the methylation mechanism of m^6^A has demonstrated that the regulation of the RNA level related to m^6^A is complex and diverse. In addition, there is unequivocal evidence that m^6^A modification is essential for a variety of biological processes, including the immune response, and there is increasing evidence that its dysregulation is linked to numerous human diseases. We believe that further research on the mechanism of m^6^A methylation in immune diseases will have profound implications for the treatment of immune diseases (**Figure 4**). New approaches and targets for the treatment of RA will certainly come to light, which will have a positive effect on human life.

## Author Contributions

SW and X-FL researched the data and drafted the manuscript. Y-YW and S-QY participated in the conception and design of the study and interpretation of the data. JL and CH reviewed the data, made substantial contributions to the discussion of the content, and edited the article before submission. All authors contributed to the article and approved the submitted version.

## Funding

This study was supported by the National Natural Science Foundation of China (No. 82002269), China Postdoctoral Science Foundation (No. 2020M671839), Postdoctoral Science Foundation from Anhui Medical University (No. BSH201902), and Anhui Provincial Science and Technology Major Project (8212929035).

## Conflict of Interest

The authors declare that the research was conducted in the absence of any commercial or financial relationships that could be construed as a potential conflict of interest.

## Publisher’s Note

All claims expressed in this article are solely those of the authors and do not necessarily represent those of their affiliated organizations, or those of the publisher, the editors and the reviewers. Any product that may be evaluated in this article, or claim that may be made by its manufacturer, is not guaranteed or endorsed by the publisher.

## Abbreviations

ALKBH5, Alkylation repair homolog protein 5; APCs, Antigen presenting cells; BC, Breast cancer; FTO, Fat mass and obesity-associated protein; HCC, Hepaticcellcarcinoma; PD-L1, programmed death-ligand 1; PDAC, Pancreatic ductal adenocarcinoma; MCs, Mast cells; METTL, Methyltransferase-like; m^6^A, *N* ^6^-methyladenosine; NK cells, Natural killer cells; RA, Rheumatoid arthritis; RBM, RNA-binding motif protein; VSMCs, Vascular smooth muscle cells; WTAP, Wilms’ tumor 1 associated protein.
